# Angiotensin III Induces JAK2/STAT3 Leading to IL-6 Production in Rat Vascular Smooth Muscle Cells

**DOI:** 10.3390/ijms20225551

**Published:** 2019-11-07

**Authors:** Ahmed Z. Alanazi, Michelle A. Clark

**Affiliations:** Department of Pharmaceutical Sciences, College of Pharmacy, Nova Southeastern University, 3200 South University Drive, Fort Lauderdale, FL 33328, USA

**Keywords:** Angiotensin III, vascular smooth muscle cells, interleukin-6, Janus kinase-2/ signal transducer and activators of transcription-3, AT_1_R

## Abstract

The Janus kinase-2/ signal transducer and activators of transcription-3 (JAK2/STAT3) pathway and interleukin-6 (IL-6) are pleiotropic signal transduction systems that are responsible for induction of many cytokines and growth factors. It is unknown whether the renin angiotensin aldosterone system (RAAS) peptide, angiotensin (Ang) III induces JAK2/STAT3 and IL-6 in vascular smooth muscle cells (VSMCs). Thus, the purpose of this study was to investigate whether Ang III induces the JAK2/STAT3 pathway leading to IL-6 production in cultured VSMCs isolated from Wistar rats and determine whether differences exist in spontaneously hypertensive rat (SHR) VSMCs. We gauged Ang III’s effects on this pathway by measuring its action on STAT3 as well as IL-6 production. Ang III behaved similarly as Ang II in stimulation of STAT3 phosphorylation in Wistar and SHR VSMCs. Moreover, there were no differences in this Ang III effect in SHR versus Wistar VSMCs. In Wistar VSMCs, Ang II and Ang III significantly induced IL-6 protein secretion and mRNA expression. However, IL-6 protein secretions mediated by these peptides were significantly greater in SHR VSMCs. Ang III induced the JAK2/STAT3 pathway, leading to IL-6 protein secretion and IL-6 mRNA expression via actions on AT_1_Rs. Moreover, the actions of Ang III to induce IL-6 production was dysregulated in SHR VSMCs. These findings suggest that Ang III acts on AT_1_Rs to induce JAK2/STAT3, leading to an increase in IL-6 in cultured VSMCs. These findings are important in establishing Ang III as an important physiologically relevant peptide in VSMCs.

## 1. Introduction

The renin-angiotensin aldosterone system (RAAS) is a peptide hormone system that contributes to various hypertensive disorders (secondary hypertensions), such as renovascular hypertension, malignant hypertension, and renin-secreting neoplasms [1]. There is evidence that RAAS dysregulation is also involved in essential hypertension since the renin plasma levels differ widely in essential hypertensive patients, and 15% of them have elevation in plasma renin activity [2]. The RAAS also has a major role in the regulation of physiological processes in the cardiovascular system [3]. The angiotensin (Ang) type 1 receptor (AT_1_R) and the Ang type 2 receptor (AT_2_R) are responsible for mediating the physiological and pathological actions of Ang II and Ang III [4,5]. These receptors generally have opposite physiological effects [6]. Interestingly, studies have confirmed that the most well-known Ang II physiological functions are mediated by AT_1_Rs, the predominant receptor in most organs [1]. Most interestingly, Ang III mediates several physiological functions in a similar manner to Ang II by activation of AT_1_Rs [7]. It is important to note that the heptapeptide Ang III [Ang-(2–8)] is produced from Ang II [Ang-(1–8)] by the enzyme aminopeptidase A [7]. Thus, the two peptides are very similar (one amino acid difference) in structure.

In our laboratory, we showed, in Wistar rat astrocytes, that Ang III induced the phosphorylation of the Janus kinase-2/ signal transducer and activators of transcription-3 (JAK2/STAT3) pathway in a concentration- and time-dependent manner, leading to astrocyte proliferation [8]. The JAK/STAT pathway is a pleiotropic signal transduction system that is involved in homeostasis, growth control and development [9,10]. This pathway is responsible for the induction of many cytokines and growth factors, including interleukin-6 (IL-6) [11,12]. It also transmits the effects of extracellular ligands through transmembrane receptors to the nucleus, leading to stimulation of genes involved in immunity, apoptosis, cell proliferation, cell migration, and differentiation [9]. These cellular actions are pivotal in many processes that encompass adipogenesis, immune development, lactation, hematopoiesis, and sexually dimorphic growth [1].

It has been proposed that Ang II induces secretion of interferon γ (IFN-γ) and IL-6, which stimulate the JAK/STAT pathway, leading to an increase in the expression of angiotensinogen in many organs and tissues. Thus, JAK/STAT promotes local Ang II synthesis and the development of hypertension as well as tissue injury in Ang II-dependent hypertension [13]. Ang II has been shown to activate JAK2 in vascular smooth muscle cells (VSMCs) [14,15]. Although the actions of Ang III on JAK2 is unknown, in our laboratory, we established that Ang III induces STAT3 phosphorylation in astrocytes [8]. Whether this action of Ang III to induce STAT3 occurs in peripheral cells, in particular, cells such as VSMCs that are known to contribute to the pathology of hypertension, is unknown and was the focus of this study. We sought to determine the role of Ang III and its molecular target STAT3 on hypertension using VSMCs isolated from Wistar rats and ascertain whether differences exist in the actions of the peptide on this target in VSMCs isolated from the spontaneously hypertensive rat (SHR). Furthermore, a number of studies indicated that IL-6 has an important role in the pathophysiology of several diseases, including hypertension, atherosclerosis, vascular occlusive disease, and vascular remodeling [16]. Moreover, overstimulation of IL-6 production can also induce pulmonary high blood pressure in mice [17]. In addition, it has been shown that the plasma levels of IL-6 are high in patients with hypertension and unstable angina [13,18]. Previously, it was shown in cultured rat VSMCs that Ang II through the JAK2/STAT3 pathway significantly increased the expression of IL-6 mRNA and IL-6 secretion in a concentration- and time-dependent manner [18,19].

However, the ability of Ang III to induce the JAK2/STAT3 pathway, leading to induction of IL-6 in VSMCs, is not known. In these studies, we determined in VSMCs whether Ang III activates the JAK2/STAT3 pathway, leading to IL-6 production. We measured the activation of STAT3 as an index of the JAK2/STAT3 pathway. 

## 2. Results

### 2.1. STAT3 Activation by Ang II and Ang III in Wistar VSMCs

**Figure 1:**
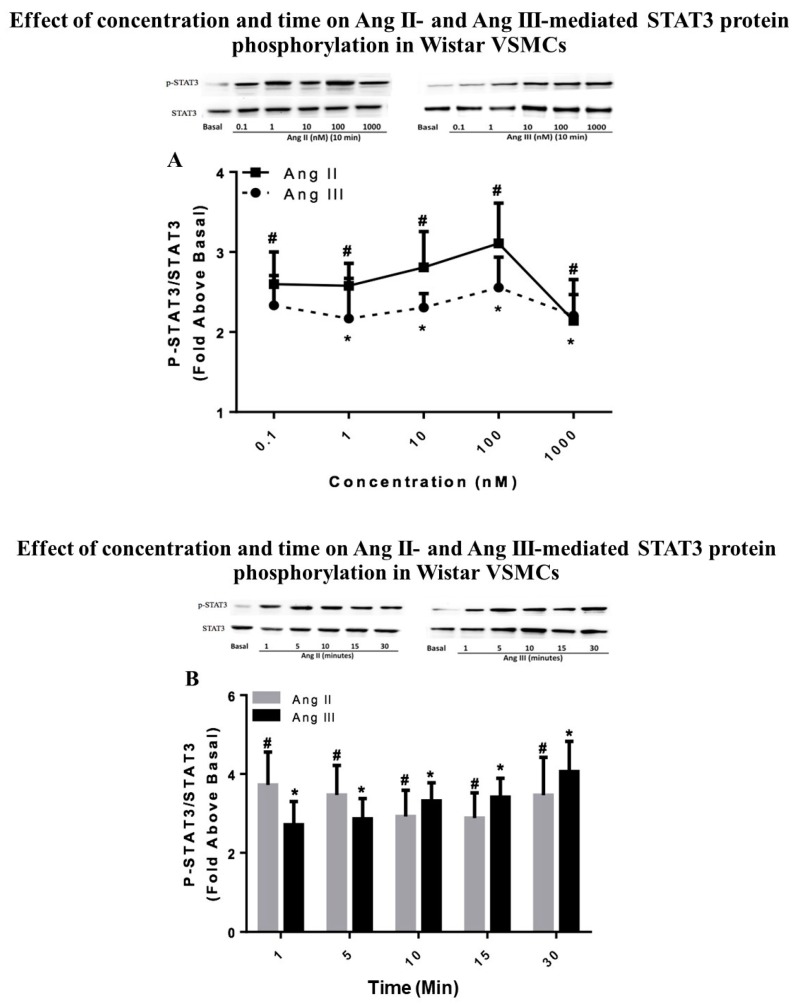
Effect of concentration and time on angiotensin (Ang) II- and Ang III-mediated STAT3 protein phosphorylation in Wistar vascular smooth muscle cells (VSMCs). Quiescent Wistar VSMCs were treated for (**A**) 10 min with either Ang II or Ang III at concentrations ranging from 0.1 nM to 1000 nM or (**B**) with 100 nM Ang II or 100 nM Ang III for 1 min to 30 min. Phosphorylated-STAT3 protein levels were measured by Western blot analysis using a specific antibody for the phosphorylated form of STAT3. Protein loading was visualized using the non-phosphorylated STAT3 antibody. The data were analyzed by densitometry and the amount of phosphorylation was calculated as the fold-increase above basal in the presence of vehicle. Each value represents the mean ± SEM of preparations of VSMCs isolated from at least five Wistar rats. * Denotes *p* < 0.05 as compared to basal levels of STAT3 expression in VSMCs for Ang III. ^#^ Denotes *p* < 0.05 as compared to STAT3 basal levels in VSMCs for Ang II.

To establish and compare the effects of Ang peptides on STAT3 protein phosphorylation in the periphery, concentration and time curves studies were performed in cultured Wistar VSMCs. The concentrations of the peptides used and the time points for treatment were selected based on previous studies [8,11,12]. Growth arrested VSMCs were incubated with increasing concentrations of Ang II and Ang III (0.1, 1, 10, 100, and 1000 nM) for 10 min to determine the concentration effects of the peptides on STAT3 phosphorylation. As demonstrated in Figure 1A, both Ang II and Ang III phosphorylated STAT3 protein in a concentration-dependent manner and the maximal stimulation was observed with 100 nM concentrations of both peptides (3.11 ± 0.50 fold above basal for Ang II versus 2.55 ± 0.38 fold above basal for Ang III). The lowest concentrations of Ang II and Ang III to cause significant phosphorylation of STAT3 protein were 0.1 nM and 1 nM, respectively, suggesting that the peptides’ effects were potent in VSMCs. Our findings indicate that both Ang II and Ang III stimulated STAT3 phosphorylation in VSMCs, and there were no statistically significant differences between their concentration effects.

In addition, the time effects of Ang peptides on stimulation of STAT3 were also observed in cultured Wistar VSMCs. Quiescent VSMCs were treated for different incubation times (1 to 30 min) with 100 nM Ang II or 100 nM Ang III. Figure 1B shows that P-STAT3 was stimulated in a time-dependent manner by both peptides. Significant induction of STAT3 phosphorylation occurred as early as 1 min after treatment with Ang II or Ang III, which continued for up to 30 min with both peptides. These findings suggest that the Ang peptides’ effects on STAT3 phosphorylation were rapid and sustained. The maximal phosphorylation of STAT3 with Ang II was observed after 1 min of treatment (3.72 ± 0.83-fold above basal), while the highest stimulation with Ang III occurred 30 min after treatment (4.06 ± 0.76-fold above basal). Although, at the later time points, the phosphorylation levels of STAT3 appeared to be higher with Ang III treatment, there were no statistically significant differences between the peptides’ effects. Our findings suggest that in VSMCs, Ang III behaves similarly as Ang II in stimulation of the STAT3 protein.

### 2.2. STAT3 Activation by Ang III in VSMCs from SHRs

**Figure 2:**
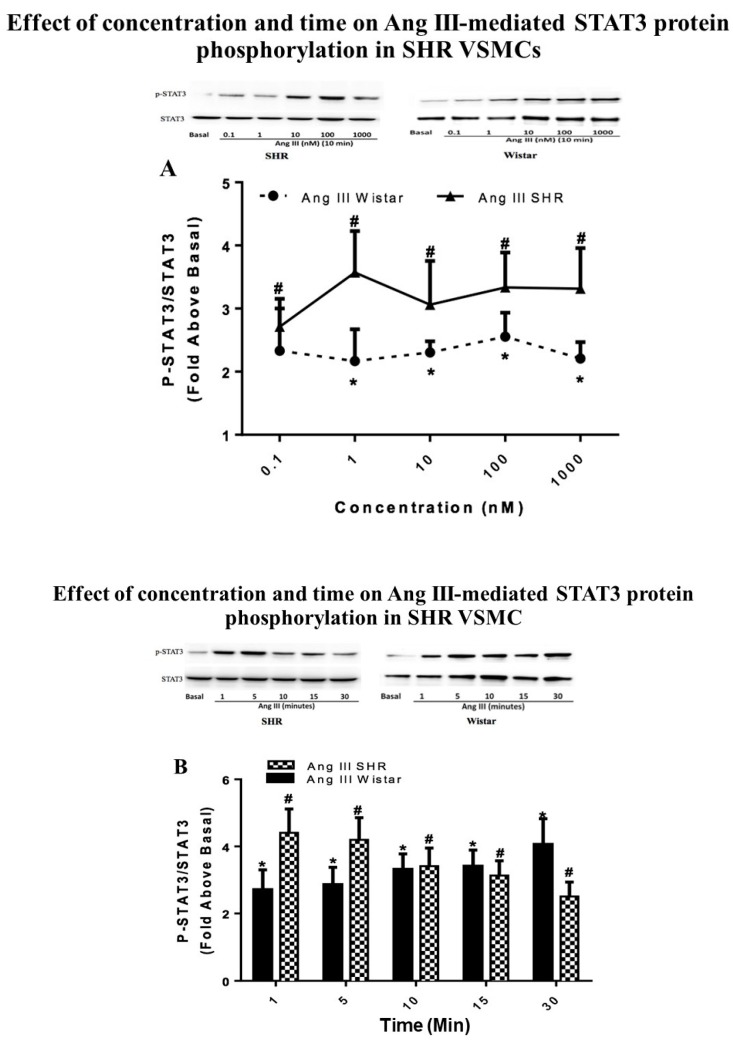
Effect of concentration and time on Ang III-mediated STAT3 protein phosphorylation in SHR VSMCs. Quiescent SHR VSMCs were treated for (**A**) 10 min with Ang III at concentrations ranging from 0.1 nM to 1000 nM or (**B**) with 100 nM Ang II or 100 nM Ang III for 1 min to 30 min. The data shown in Figure 2A,B are the same as the data in Figure 1A,B, respectively, for the Wistar Ang III data. Phosphorylated-STAT3 protein levels were measured by Western blot analysis using a specific antibody for the phosphorylated form of STAT3. Protein loading was visualized using the non-phosphorylated STAT3 antibody. The data were analyzed by densitometry and the amount of phosphorylation was calculated as the fold-increase above basal in the presence of vehicle. Each value represents the mean ± SEM of preparations of VSMCs isolated from at least five Wistar or SHRs. * Denotes *p* < 0.05 as compared to basal levels of STAT3 expression in VSMCs isolated from Wistar rats. ^#^ Denotes *p* < 0.05 as compared to STAT3 basal levels in VSMCs isolated from SHRs.

In the current study, the effect of Ang III on STAT3 phosphorylation was also investigated in SHR VSMCs and the results were also compared with those obtained from Wistar VSMCs. Quiescent VSMCs isolated from SHRs were treated with Ang III ranging in concentrations from 0.1 nM to 1000 nM or with 100 nM Ang III for 1 to 30 min to determine the effect of concentration and time on Ang III-induced STAT3 protein phosphorylation. As shown in Figure 2, Ang III phosphorylated STAT3 protein in a concentration- and time-dependent manner in SHR VSMCs. The maximal stimulation was observed with 1 nM Ang III (3.57 ± 0.65-fold above basal) as well as between 1 and 5 min of Ang III treatments (4.40 ± 0.71-fold above basal). Significant phosphorylation of STAT3 by Ang III occurred as early as 1 min and at Ang III concentrations as low as 0.1 nM. These findings suggest that Ang III-induced phosphorylation of the STAT3 protein was rapid, sustained, and potent in SHR VSMCs.

The comparison between the effects of Ang III in SHR versus Wistar VSMCs shows no statistically significant differences in the abilities of VSMCs isolated from these two models to respond to the peptide. Moreover, the basal levels of STAT3 were similar in Wistar VSMCs and SHR VSMCs (0.3798 ± 0.06 arbitrary unit versus 0.3408 ± 0.04 arbitrary unit; *p* = 0.569 in Wistar versus SHR VSMCs, respectively).

### 2.3. Effects of AG490 and Ang Receptor Blockers on Ang III-induced STAT3 Phosphorylation

**Figure 3:**
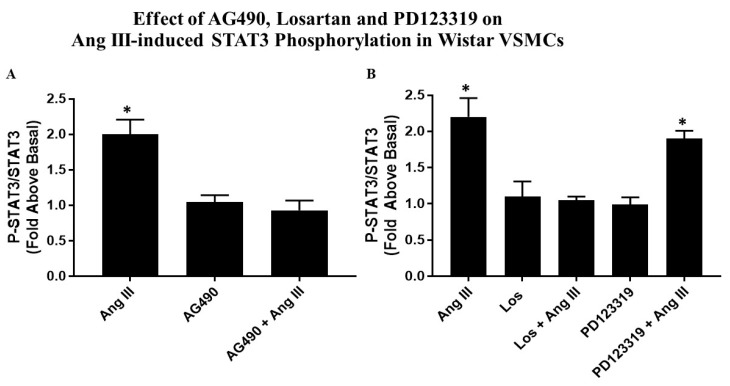
Effects of AG490, Losartan, PD123319 on Ang III-induced STAT3 Phosphorylation in Wistar VSMCs. Quiescent Wistar VSMCs were pretreated with either 50 µM AG490 (**A**) or 10 µM Losartan (Los) or 10 µM PD123319 (**B**) for 15 min. Subsequently, the cells were treated with or without 100 nM Ang III for 10 min. Phosphorylated-STAT3 protein levels were measured by Western blot analysis using a specific antibody for the phosphorylated form of STAT3. Protein loading was visualized using the non-phosphorylated STAT3 antibody. The data were analyzed by densitometry and the amount of phosphorylation was calculated as the fold-increase above basal in the presence of vehicle. The vehicle for AG490 was DMSO; incubation with Ang III was also done in the presence of DMSO. Each value represents the mean ± SEM of preparations of VSMCs isolated from at five or more Wistar rats. * Denotes *p* < 0.05 as compared to basal levels of STAT3 expression in VSMCs isolated from Wistar rats.

Quiescent VSMCs were preincubated with the JAK2 inhibitor (AG490, 50 µM), or with 10 µM Losartan (AT_1_R blocker) or with 10 µM PD123319 (AT_2_R blocker) for 15 min followed by 100 nM Ang III for 10 min. Co-treatment of VSMCs with AG490 and Ang III completely abolished all of Ang III’s effect on P-STAT3. Treatment with AG490 alone had no effect on basal P-STAT3 (Figure 3A).

As shown in Figure 3B, the AT_1_ R blocker completely inhibited this Ang III effect; however, the AT_2_R blocker was ineffective in preventing Ang III-mediated STAT3 phosphorylation. These results indicate that Ang III interacts with the AT_1_ receptor to directly phosphorylate STAT3 protein in VSMCs.

### 2.4. Effects of Ang II and Ang III on Wistar VSMC IL-6 Secretion

**Figure 4:**
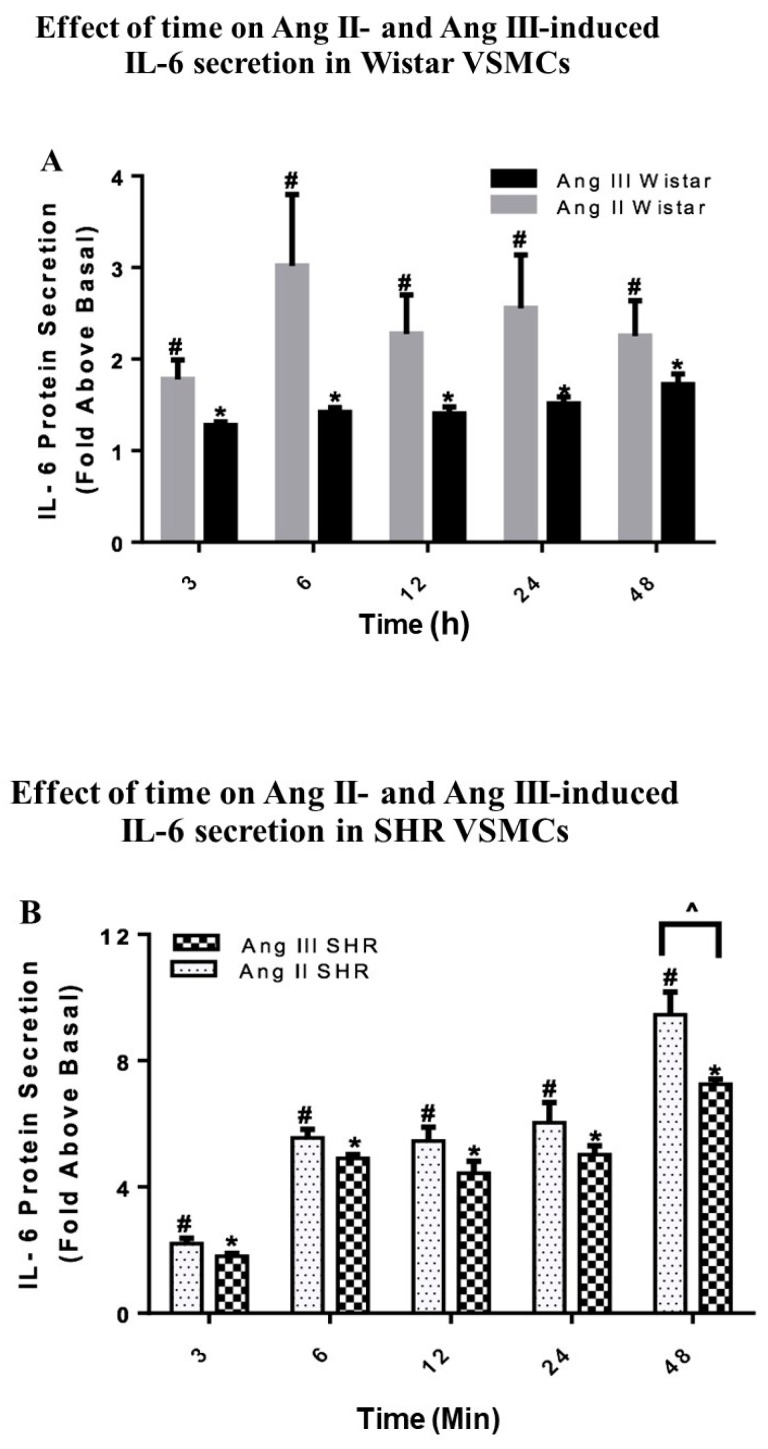

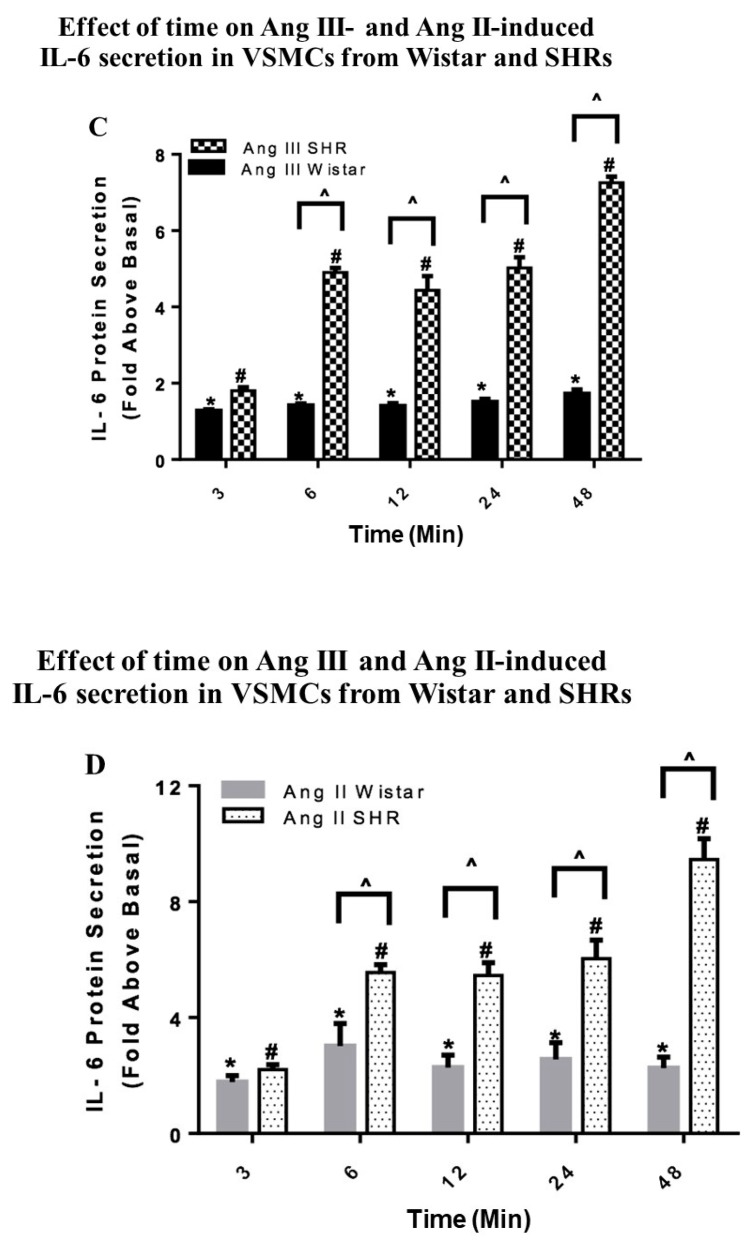
Effect of time on Ang II- and Ang III-induced IL-6 secretion in Wistar and SHR VSMCs. Quiescent VSMCs isolated from (**A**) Wistar rats treated with Ang II or Ang III, and (**B**) SHRs treated with Ang II or Ang III were treated with 100 nM Ang II or 100 nM Ang III for 3 h to 48 h. Panel (**C**) compares Ang III-mediated IL-6 secretion from Wistar and SHR VSMCS. Panel (**D**) compares Ang II-mediated IL-6 secretion from Wistar and SHR VSMCs. Secreted levels of IL-6, induced by Ang II and Ang III, were calculated as the fold above basal and were analyzed by an ELISA method. Each value represents the mean ± SEM of preparations of VSMCs isolated from four or more SHRs or Wistar rats. * Denotes *p* < 0.05 as compared to basal levels of IL-6 secretion in VSMCs treated with Ang III. ^#^ Denotes *p* < 0.05 as compared to basal levels of IL-6 secretion in VSMCs treated with Ang II. ^ Denotes *p* < 0.05 as compared between the effects of Ang II or Ang III in stimulation of IL-6 secretion in VSMCs isolated from SHRs versus Wistar rats.

To determine whether Ang III is analogous to Ang II in stimulation of IL-6 protein secretion in VSMCs, growth arrested VSMCs isolated from Wistar rats were treated with 100 nM Ang II or 100 nM Ang III for time periods ranging from 3 h to 48 h. The peptide concentrations and the time course for peptide treatments were selected based on previous studies [11]. Then, the enzyme linked immunosorbent assay (ELISA) method was used to determine the amount of IL-6 secreted in the media. As shown in Figure 4A, both Ang peptides stimulated IL-6 secretion in a time-dependent manner. Significant stimulation of IL-6 secretion by Ang II and Ang III occurred after 3 h of treatment and maximal effects were observed 6 h after Ang II treatment and 48 h after Ang III treatment (3.01 ± 0.77-fold above basal with Ang II and 1.72 ± 0.11-fold above basal with Ang III). Although there was a tendency for higher IL-6 secretion from Wistar VSMCs treated with Ang II, there were no significant differences between Ang II- and Ang III-mediated IL-6 secretion.

### 2.5. Effects of Ang II and Ang III on SHR VSMC IL-6 Secretion

VSMCs isolated from SHRs were treated in a similar manner as Wistar VSMCs. Figure 4B shows that Ang II and Ang III induced IL-6 secretion in a time-dependent manner with maximal effects observed by 48 h after treatment with both peptides. There was a gradual and significant increase in IL-6 production at all-time points measured. Interestingly, the differences between the effects of Ang II and Ang III in SHR VSMCs were significant only after 48 h of treatment (9.45 ± 0.71-fold above basal with Ang II versus 7.25 ± 0.16-fold above basal with Ang III).

In comparison to the findings in Wistar VSMC, the secreted levels of IL-6 by the peptides were significantly greater in SHR VSMCs at all the time points measured except for the 3 h time point (Figure 4C,D). Most importantly, the basal secretion of IL-6 were similar (*p* = 0.147) in Wistar versus SHR VSMCs (0.08 ± 0.004 versus 0.15 ± 0.037, respectively). These findings indicate that IL-6 secretion mediated by Ang peptides is different in the hypertensive state as SHR VSMCs ability to secrete IL-6 is more sensitive to the Ang peptides as compared to the peptides’ actions in Wistar VSMCs.

### 2.6. Effects of AG490, Losartan and PD123319 on Ang III-induced IL-6 Secretion

**Figure 5:**
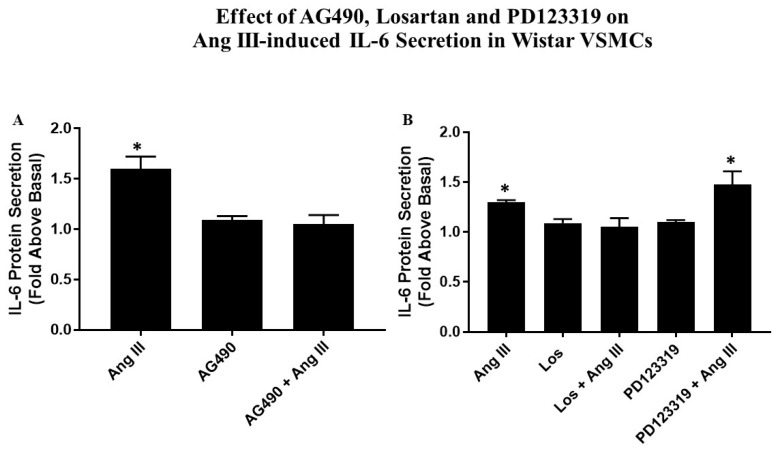
Effects of AG490, Losartan, PD123319 on Ang III-induced IL-6 secretion in Wistar VSMCs. Quiescent Wistar VSMCs were pretreated with either 50 µM AG490 (**A**) or 10 µM Losartan (Los) or 10 µM PD123319 (**B**) for 15 min. Subsequently, the cells were treated with or without 100 nM Ang III for 3 h. Secreted levels of IL-6, induced Ang III from Wistar VSMCs, were calculated as the fold above basal and were analyzed by an ELISA method. The vehicle for AG490 was DMSO; incubation with Ang III was also done in the presence of DMSO. Each value represents the mean ± SEM of preparations of VSMCs isolated from four or more Wistar rats. * Denotes *p* < 0.05 as compared to basal levels of IL-6 secretion from VSMCs isolated from Wistar rats.

Quiescent VSMCs were pretreated with 50 µM AG490 (the JAK2 inhibitor) or 10 µM Losartan (AT_1_R antagonist) or 10 µM PD123319 (AT_2_R antagonist) followed by 100 nM Ang III for 3 h. Then, ELISA was employed to measure the secreted levels of IL-6. Pretreatment with AG490 completely abolished Ang III-induced IL-6 secretion (Figure 5A). Further Losartan completely blocked Ang III-mediated IL-6 protein secretion, but PD123319 had no significant effect (Figure 5B). These findings suggest that via AT_1_Rs, Ang III increases IL-6 protein secretion, an effect that was mediated by JAK2.

### 2.7. Effects of Ang II and Ang III on IL-6 mRNA Expression in Wistar VSMCs

**Figure 6:**
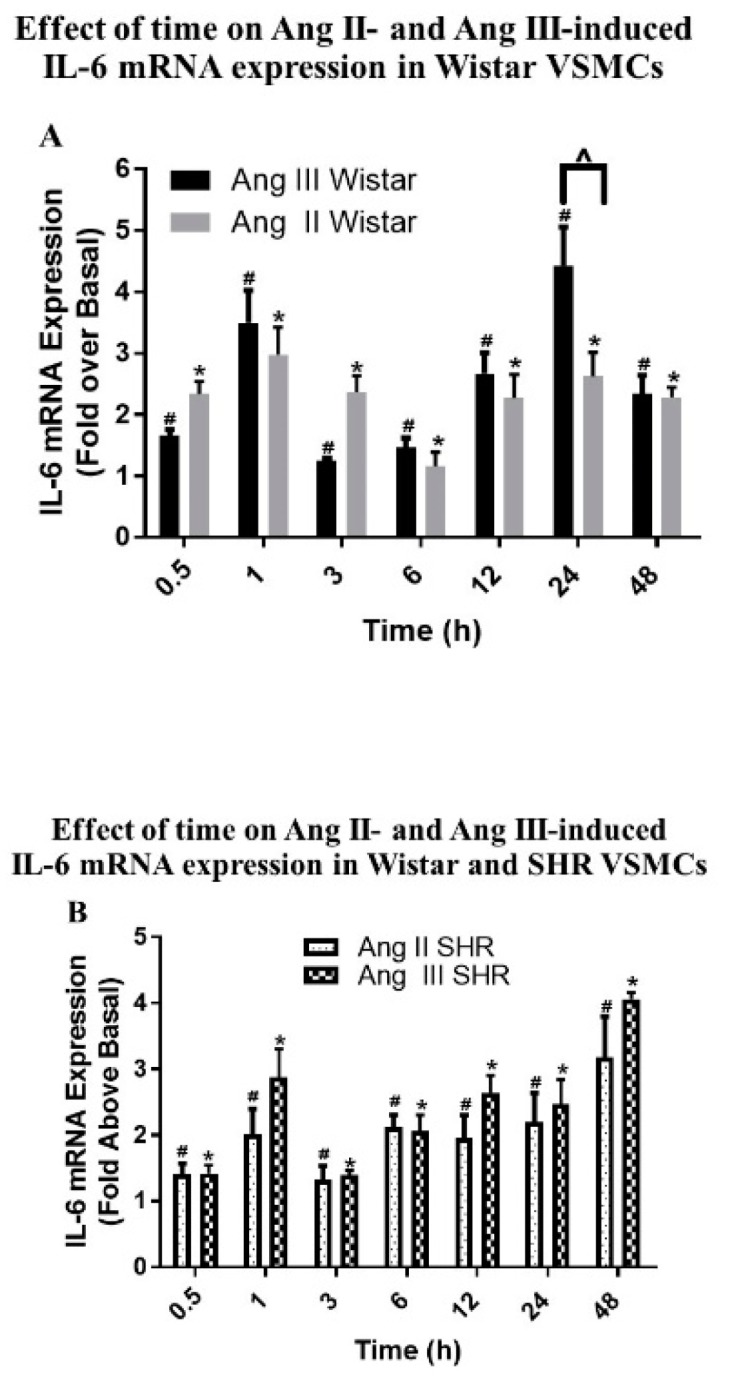

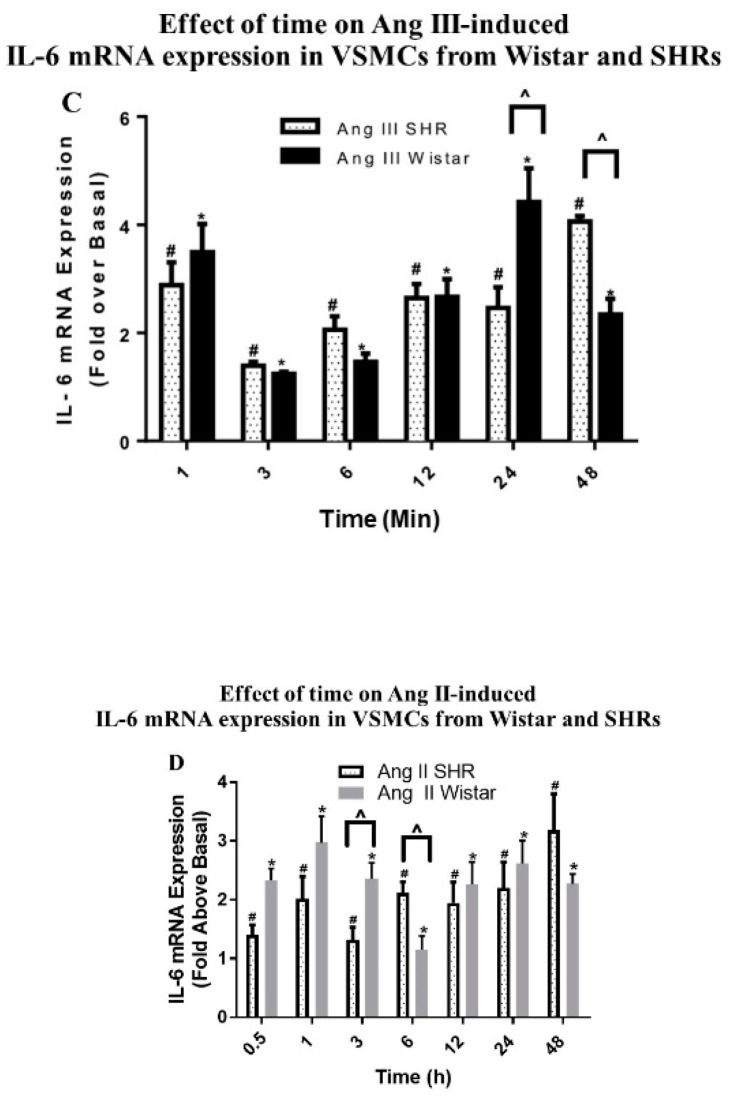
Effect of time on Ang II- and Ang III-induced IL-6 mRNA expression in Wistar and SHR VSMCs. Quiescent VSMCs isolated from (**A**) Wistar rats treated with Ang II or Ang III, and (**B**) SHRs treated with Ang II or Ang III were incubated with 100 nM Ang II or 100 nM Ang III for 1 h to 48 h. Panel (**C**) compares Ang III-mediated IL-6 mRNA expression from Wistar and SHR VSMCS. Panel (**D**) compares Ang II-mediated IL-6 mRNA expression from Wistar and SHR VSMCs. IL-6 mRNA expression was analyzed by quantitative PCR. The amount of Ang II- and Ang III-stimulated IL-6 mRNA expression was calculated as the fold-increase above basal. Each value represents the mean ± SEM of preparations of VSMCs isolated from four or more Wistar rats or SHRs. * Denotes *p* < 0.05 as compared to basal levels of IL-6 mRNA expression in Wistar VSMCs treated with Ang III or Ang II. # Denotes *p* < 0.05 as compared to basal levels of IL-6 mRNA expression in SHR VSMCs treated with Ang II or Ang III. ^ Denotes *p* < 0.05 as compared between the effects of Ang II or Ang III in stimulation of IL-6 mRNA expression in VSMCs isolated from Wistar rats versus SHRs.

In these studies, the roles of Ang II and Ang III in mediating the expression of IL-6 mRNA were determined and compared using qPCR as previously described [12]. Growth arrested VSMCs isolated from Wistar rats and SHRs were incubated with 100 nM Ang II or 100 nM Ang III for various time periods ranging from 0.5 to 48 h. As shown in Figure 6A, the Ang peptides upregulated IL-6 mRNA levels in a time-dependent manner in Wistar VSMCs. The maximal effect of Ang II occurred at 1 h (2.97 ± 0.44-fold above basal), while the highest stimulation of IL-6 mRNA expression by Ang III was 4.42 ± 0.62-fold above basal after 24 h of treatment. Interestingly, both peptides increased the expression of IL-6 mRNA levels in a biphasic fashion. The first peak was observed after 1 h of treatment with the Ang peptides and the second peak was observed after 24 h of treatment with Ang II or Ang III.

Comparing the Ang peptides’ effects on IL-6 mRNA expression revealed that there were no statistically significant differences between Ang II- and Ang III-induced IL-6 mRNA levels in Wistar VSMCs, except at the 24 h treatment point where the expression of IL-6 mRNA induced by Ang III was higher than the Ang II effect (4.42 ± 0.62-fold above basal versus 2.61 ± 0.39-fold above basal). Our findings suggest that Ang III, like Ang II, has a pro-inflammatory effect in VSMCs, as assessed by the increases observed in IL-6 mRNA expression caused by the peptide.

### 2.8. Effects of Ang II and Ang III on IL-6 mRNA Expression in SHR VSMCs

As shown in Figure 6B in SHR VSMCs, IL-6 mRNA levels induced by Ang II and Ang III were increased time-dependently and in a biphasic manner. The first peak was observed after 1 h of treatment with the Ang peptides, while the second peak of the effects of Ang III and Ang II was seen at 48 h and 24 h of treatments, respectively.

The highest effects of Ang II and Ang III in SHR VSMCs occurred after 48 h of treatment (3.2 ± 0.6 and 4.1 ± 0.1-fold above basal, respectively). The basal IL-6 cycle threshold (Ct) values were not significantly different in the Wistar (25.6 ± 0.8) as compared to SHR samples (25 ± 0.6; *p* = 0.537). These findings indicate that Ang II and Ang III have similar potencies to stimulate IL-6 mRNA levels in SHR VSMCs.

### 2.9. Effects of Ang III in Wistar versus SHR VSMCs

In comparison to the finding in Wistar VSMCs, Ang III-increased IL-6 mRNA expression was statistically higher in Wistar as compared to SHR VSMCs only after 24 h of treatment (4.42 ± 0.62-fold above basal versus 2.46 ± 0.38-fold above basal) (Figure 6C). After 48 h of treatment, the effects in SHR VSMCs increased while the effects of Ang III in Wistar VSMCs diminished and the difference was statistically significant (Figure 6C). On the other hand, the effect of Ang II on SHR VSMCs IL-6 mRNA expression levels as compared to Wistar VSMCs was different at earlier time points where IL-6 mRNA expression levels were significantly higher in Wistar versus SHR VSMCs after 3 h of treatment (2.35 ± 0.27 fold above basal versus 1.32 ± 0.21 fold above basal). However, after 6 h of treatment, IL-6 mRNA expression levels were statistically greater in SHR versus Wistar VSMCs (2.12 ± 0.18-fold above basal versus 1.15 ± 0.23-fold above basal) (Figure 6D). These findings suggest that the peptides had differential effects to induce IL-6 mRNA levels as the Ang III-mediated IL-6 mRNA expression was higher at the later time points of treatment while the effect of Ang II at the earlier time points of treatment were higher in Wistar than SHR VSMCs.

### 2.10. Effects of AG490, Losartan and PD123319 on Ang III-induced IL-6 mRNA Expression

**Figure 7:**
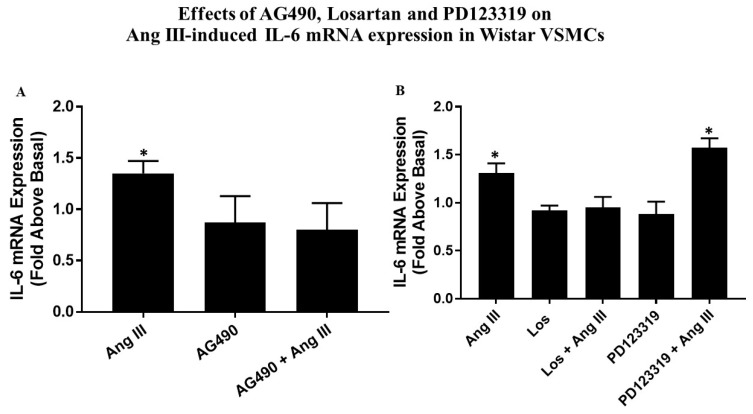
Effects of AG490, Losartan, PD123319 on Ang III-induced IL-6 mRNA expression in Wistar VSMCs. Quiescent Wistar VSMCs were pretreated with either 50 µM AG490 (**A**) or 10 µM Losartan (Los) or 10 µM PD123319 (**B**) for 15 min. Subsequently, the cells were treated with or without 100 nM Ang III for 3 h. IL-6 mRNA expression was analyzed by quantitative PCR. The vehicle for AG490 was DMSO; incubation with Ang III was also done in the presence of DMSO. Each value represents the mean ± SEM of preparations of VSMCs isolated from four or more Wistar rats. * Denotes *p* < 0.05 as compared to basal levels of IL-6 mRNA expression from VSMCs isolated from Wistar rats.

Growth arrested VSMCs were preincubated with 50 µM AG490, 10 µM Losartan, or 10 µM PD123319 and then treated with 100 nM Ang III for 3 h. Figure 7B demonstrates that PD123319, the AT_2_R blocker, did not prevent Ang III-mediated IL-6 mRNA expression, while Losartan, the AT_1_R inhibitor, blocked Ang III-mediated IL-6 mRNA expression by 100%. The presence of AG490 (Figure 7A) also abolished the induction of Il-6 mRNA expression mediated by Ang III. These findings suggest that via AT_1_Rs, Ang III increases IL-6 mRNA expression, an effect that involved the JAK2 pathway in VSMCs.

## 3. Discussion

In previous studies, we have established the roles of Ang III in cultured astrocytes isolated from neonatal rat pups. We demonstrated that Ang III, similarly to Ang II, stimulated mitogen activated protein kinases (MAPKs) as well as the JAK2/STAT3 signaling pathways, leading to astrocyte proliferation [8,12,20,21,22,23]. Additionally, Ang III increased IL-6 secretion and the expression of IL-6 mRNA through activation of the JAK2/STAT3 cascade, suggesting pro-inflammatory effects of the peptide [8]. Most importantly, Ang III induced these effects via interaction with AT_1_Rs. It is well known that the JAK2/STAT3 signaling pathway mediates the stimulation of different cytokines and growth factors that regulate multiple cell functions, including proliferation, apoptosis, survival, and differentiation [9,10]. As mentioned, we established a role for Ang III as a mediator of this pathway leading to pro-inflammatory responses in the central nervous system, specifically in astrocytes. However, the role of Ang III to affect the JAK2/STAT3 signaling pathway in VSMCs was unknown and the focus of this study. Specifically, we measured basal STAT3, and the ability of Ang III to induce STAT3 activation as a measure of the JAK2/STAT3 pathway in normotensive (Wistar) and hypertensive (SHR) VSMCs.

We found that Ang III was just as potent as Ang II in stimulating STAT3 phosphorylation. Surprisingly, there were no differences in the ability of Ang III to induce STAT3 phosphorylation in Wistar versus SHR VSMCs, and the basal levels of STAT3 were similar in Wistar and SHR VSMCs. The finding that Ang III is a potent inducer of STAT3 in these cells is similar to our previous findings in Wistar astrocytes [8]. In astrocytes, the concentration effect of Ang III to induce STAT3 phosphorylation was similar with the greatest effect occurring with 100 nM Ang III [8]. Furthermore, the actions of Ang II on STAT3 phosphorylation was also similar to the actions of the peptide in primary cultured brainstem astrocytes [11]. These findings suggest that under these conditions and in this cell type, there was no dysregulation in basal or Ang III-mediated levels of STAT3.

Experimental evidence from both in vivo and in vitro studies established that activation of the JAK2/STAT3 pathway by Ang II plays a central role in the development of Ang II-dependent hypertension [13]. Recent studies have shown that Ang II induced undesirable elevation of various pro-inflammatory factors and cytokines that in turn activate the JAK2/STAT3 signaling pathway, leading to activation of intrarenal RAAS. This led to the establishment of renal injury and the progression of high blood pressure [13]. In VSMCs, stimulation of JAK2 by Ang II leads to activation of RhoA guanine nucleotide exchange factor I, Arhgef1, which, in turn, stimulates RhoA signaling, causing hypertension [24]. On the other hand, inactivation of Arhgef1 attenuates Ang II-mediated high blood pressure. Furthermore, it was shown that pharmacological inhibition of JAK2 diminished the development of hypertension in Ang II-infused animals [25]. This effect has also been observed in JAK2 knockdown cells [26]. In vivo studies also showed that inhibition of JAK2 and AT_1_Rs significantly reduced the development of both hypertension and proteinuria in diabetic nephropathy [13,27]. Ang II caused biphasic STAT3 activation in cardiomyocytes, an effect that was mediated by IL-6 [28]. These findings suggest a crucial role of JAK2/STAT3 in the development of Ang II-mediated hypertension. We did not find differences in Ang III-mediated STAT3 phosphorylation in SHR VSMCs as compared to the effects observed in Wistar VSMCs. As hypertension is a multi-organ disease, it is plausible that this particular pathway is unaffected in VSMCs obtained from this hypertensive model. Moreover, we measured STAT3 as an index of the JAK2/STAT3 pathway. It is possible that the STAT3 arm of the pathway was unaffected under these conditions.

It has been reported that Ang II, the primary peptide of the RAAS, exerts a number of actions in VSMCs that include vascular inflammation, vasoconstriction, VSMC proliferation, generation of growth factors and cytokines, as well as vasomotor tone modulation [3,13]. Furthermore, studies showed that Ang II increased the expression of tissue and plasma pro-inflammatory cytokines such as IL-6, IL-1β, IFN-γ, and TNFα [13]. Moreover, in vascular tissues, Ang II, via activation of AT_1_Rs, causes accumulation of inflammatory mediators, fibrosis and VSMC migration and growth, suggesting a crucial role of Ang II in mediating vessel wall inflammation and endothelium dysfunction [16,29,30]. In VSMCs isolated from humans and rats, IL-6 is secreted in response to Ang II and TNF [31,32]. Funakoshi et al. showed that Ang II, via AT_1_Rs, significantly increased the expression of IL-6 mRNA and protein levels in a biphasic, time- and concentration-dependent manner in cultured rat VSMCs [18]. They suggested that stimulation of IL-6 expression by Ang II in VSMCs was dependent on the activation of MAPKs, tyrosine phosphorylation and intracellular Ca^2+^.

In the current study, we determined the actions of Ang III on IL-6 secretion and mRNA levels in Wistar and SHR VSMCs. Similarly, Ang II and Ang III induced IL-6 levels in a biphasic manner. We have found similar actions (including the biphasic effect) of Ang II in astrocytes [8,11,12]; others have found the biphasic release and synthesis of IL-6 by Ang II in VSMCs [18]. This Ang III action was mediated by the AT_1_R and dependent on activation of the JAK2/STAT3 pathway. This finding in VSMCs corroborated our findings in rat astrocytes [11]. Most importantly, the secretion of IL-6 from SHR VSMCs by both peptides was significantly higher at most time points examined, suggesting an important difference in the response of these cells to the actions of the peptides. The findings in the effects of the peptides on IL-6 mRNA were not as striking as, at most time points examined, the effects of the peptides in Wistar and SHR VSMCs were similar. The reason for this discrepancy is unclear but may reflect multiple and complicated post-translational mechanisms involved in converting mRNA into proteins that are not defined completely enough to calculate protein concentrations from mRNA. Furthermore, the in vivo half-lives of proteins may differ substantially than what is presumed [33,34,35]. As the protein levels of IL-6 reflects the active physiological moiety, the Ang III-mediated increase in secreted IL-6 from VSMCs is relevant in establishing the pro-inflammatory nature of the peptide. Furthermore, this Ang III action is mediated by JAK2/STAT3 and is significantly higher in SHR VSMCs. These findings suggest that an inflammatory environment may be in existence in VSMCs from this hypertensive model and corroborates with previous findings of increased levels of IL-6 in hypertension [13,18].

In conclusion, the current study provided insight into the mechanisms underlying the actions of Ang III in the periphery, specifically in VSMCs. Furthermore, we reported that Ang III behaved in an analogous manner as Ang II to activate STAT3 signaling and to induce IL-6 levels in cultured VSMCs. As far we know, this is the first report showing, in rat aortic smooth muscle cells, that Ang III is pro-inflammatory because it acts on AT1Rs leading to IL-6 secretion, an effect that is mediated by the JAK2/STAT3 pathway. Most importantly, we established that Ang III differentially induced IL-6 levels in SHR VSMCs. As most studies elucidating the actions of Ang III have occurred in the central nervous system, with little to no studies establishing the role of this peptide in VSMCs, these findings lend credence to the importance of Ang III as a physiologically relevant peptide in the cardiovascular system, in particular in VSMCs.

## 4. Materials and Methods

### 4.1. Materials

Ang III and Ang II were purchased from Bachem (Torrance, CA, USA). Tissue culture supplies, such as fetal bovine serum (FBS), Dulbecco’s modified Eagles Medium (DMEM)/F12 (1:1), streptomycin, penicillin and trypsin/ethylenediamine tetraacetic acid (EDTA) were purchased from Fisher Scientific (Milford, MA, USA) or VWR (Suwannee, GA, USA). The AT_1_R blocker (Losartan) was provided by Du Pont Merck (Wilmington, DE, USA). The AT_2_R blocker (PD123319), and the JAK2 inhibitor (AG490) were purchased from Sigma-Aldrich (St. Louis, MO, USA). Protein measurement supplies, acrylamide, enhanced chemiluminescence (ECL) reagents, nitrocellulose membrane, gel electrophoresis and Western blotting supplies inclusive of bicinchoninic acid (BCA) protein reagents were purchased from either Bio-Rad Laboratories (Hercules, CA, USA) or VWR (Piscataway, NJ, USA). The non-phosphorylated and phospho-specific STAT3 antibodies were obtained from Cell Signaling Technology (Beverly, MA, USA). Applied Biosystems (Foster City, CA, USA) supplied reverse transcription reagent kit and TaqMan gene expression primers for rat IL-6. An ELISA kit for IL-6 was purchased from R&D Systems (Minneapolis, MN, USA). All the other chemicals were obtained from either Sigma-Aldrich (St. Louis, MO, USA) or Fisher Scientific (Milford MA, USA) or VWR international (Suwannee, GA, USA).

### 4.2. Isolation and Culture of Primary VSMCs

Male Wistar rats and SHRs (averaged blood pressure 172/123), weighing 101–135 g and 36–40 days old were acquired from Charles River Laboratories (Wilmington, MA, USA) after the protocols were approved by Nova Southeastern University Institutional Animal Care and Use committee. These rats were maintained in the ALAAC accredited animal facility of Nova Southeastern University. All animal protocols complied with the ethical treatment of animals as outlined in the NIH guide for animal care and use. The primary cultures of VSMCs were isolated from the thoracic aorta of adult Wistar rats and SHRs by the explant technique as described [36,37]. During the VSMC isolation procedure, care was taken to lessen pain and discomfort to the animals.

Carbon dioxide (CO_2_) was used to anesthetize the rats. The aorta were extracted and placed in 100 mm sterile culture plates that contained sterile phosphate buffer saline (PBS: 0.5 mM KH_2_PO_4,_ 3.2 mM Na_2_HPO_4_, 135 mM NaCl, 1.3 mM KCl, pH 7.4). Aorta was cleaned from coagulated blood and fat tissues and slit (top to bottom) and chopped into one-millimeter pieces (strips). The strips were placed into 100 mm culture plates and covered with DMEM/F12 (containing streptomycin (100 units/mL), penicillin (100 μg/mL) and 20% FBS (fetal bovine serum). These culture plates were kept in a humidified incubator (95% air and 5% CO_2_) at 37 ºC until enough VSMCs grew from the strips. The cells were passaged to T-75 flasks and allowed to proliferate to provide ample cells to perform the proposed experiments. All treatments were performed on quiescent (growth arrested) cells. Cells were made quiescent by incubating in serum free media (DMEM/F12 culture media contained; 100 units’/mL streptomycin and 100 μg/mL penicillin) for 24 to 48 h prior to treatment.

### 4.3. Cell Lysate Preparation

Immediately after VSMC treatments, cell lysates were prepared by washing the cells with PBS containing NaVO_4_ (0.01 mM). Supplemented lysis buffer (50 mM NaF, 100 mM NaCl, 1% Triton X-100, 5 mM EDTA, 50 mM Tris-HCl, 0.1 mM PMSF, 0.01 mM NaVO_4_ and 0.6 μM leupeptin, pH 7.4) was used to solubilized VSMCs, for 30 min on ice. Supernatant was clarified by centrifugation at 12000 rpm for 10 min at 4 °C and the bicinchoninic acid (BCA) method was used to measure the lysate protein concentrations according to the instructions of the manufacturer (Pierce Biotechnology, Rockford, IL, USA).

### 4.4. Western Blotting

Electrophoresis was used to separate solubilized proteins (20 μg) on 10% sodium dodecyl sulfate (SDS) polyacrylamide gels and transferred to nitrocellulose membranes; subsequently, the membranes were blocked by incubation with 5% Botto (1% Tween in Tris-buffered saline; 5% evaporated milk) to prevent nonspecific antibody binding to the membranes. Consequently, the membranes were incubated overnight at 4 °C with specific primary antibodies. Subsequently, this was followed by the addition of goat anti-rabbit secondary antibody coupled to horseradish peroxidase for 1 h at room temperature (25 °C). Immunoreactive bands were visualized using ECL reagents.

### 4.5. Total RNA Extraction and Measurement of IL-6 mRNA Levels

The trizol method was used to extract total RNA from VSMCs. Subsequently, the total RNA was subjected to a DNA clean up step before the concentrations of total RNA was measured using a Bio-Rad SmartSpecTM spectrophotometer (Bio-Rad Laboratories). Total RNA (2 μg) was reverse transcribed to complementary strand DNA using a high capacity reverse transcription reagent kit (Applied Biosystems, Foster City, CA, USA). Quantitative PCR (qPCR) was performed using the TaqMan Universal master mix, and the TaqMan gene expression primers for rat IL-6 (Rn01410330; Applied Biosystems, Foster City, CA, USA). Samples were analyzed in 96-well plates using the StepOneTM plus a real-time PCR system (Applied Biosystems, Foster City, CA, USA). To perform relative quantification of qPCR, the comparative Ct (threshold cycle) method was used [38]. After normalizing to levels of the housekeeping control gene beta actin (Rn00667869; Applied Biosystems, Foster City, CA, USA), the relative IL-6 mRNA expression in Ang II- or Ang III-treated VSMCs over the untreated controls were calculated for each target gene using an arithmetic formula (fold difference = 2^−ΔΔCt^).

### 4.6. Enzyme-Linked Immunosorbent Assay

An ELISA kit for IL-6 was used for the quantitative measurement of IL-6 that was secreted in VSMCs culture media. The measurements were performed according to the manufacture’s protocol (R&D Systems, Minneapolis, MN, USA).

Samples that contained IL-6 protein were added to the 96-well microplates. After the antigen was immobilized, another specific antibody against rat IL-6, conjugated to horseradish peroxidase, was added. The fluorescent signal was generated by horseradish peroxidase enzyme. Finally, this fluorescent signal was measured using a Biotek microplate reader to determine the presence and quantity of IL-6 in the samples.

### 4.7. Statistical Analysis

The data are expressed as mean ± SEM. Repeated measures one-way analysis of variance (ANOVA) followed by Dunnetts’s post hoc test or t-tests were employed to juxtapose/compare the groups. The comparison between the effects in Wistar versus SHRs was carried out using two-way ANOVA with Tukey’s test. Statistical significance was set at *p* < 0.05.

## Abbreviations

MDPI	Multidisciplinary Digital Publishing Institute
Ang	Angiotensin
AT_1_R	angiotensin type I receptor
AT_2_R	angiotensin type 2 receptor
ANOVA	analysis of variance
BCA	bicinchoninic acid
DMEM/F12	Dulbecco’s modified Eagles Medium/F12
DMSO	dimethyl sulfoxide
ECL	enhanced chemiluminescence
EDTA	ethylenediamine tetraacetic acid
ELISA	enzyme linked immunosorbent assay
FBS	fetal bovine serum
INF	interferon
IL-	interleukin
JAK2/STAT3	Janus kinase-2/ signal transducer and activators of transcription-3
MAPK	mitogen activated protein kinase
PBS	phosphate buffer saline
qPCR	quantitative PCR
RAAS	renin angiotensin aldosterone system
SDS	sodium dodecyl sulfate
SHR	spontaneously hypertensive rat
VSMC	vascular smooth muscle cell
